# Positive, Neutral, or Just Normal? A Focus Group Study on Adolescents’ Knowledge, Familiarity and Perceived Effectiveness of Body Positivity and Body Neutrality on Social Media

**DOI:** 10.3390/bs16050680

**Published:** 2026-04-29

**Authors:** Gaëlle Ouvrein, Lara Schreurs

**Affiliations:** 1 Studies in Media, Innovation and Technology (SMIT), Department Communication Studies, Vrije Universiteit Brussel (VUB), 1050 Ixelles, Belgium; 2 Media Psychology Lab, Faculty of Social Sciences, KU Leuven, 3000 Leuven, Belgium

**Keywords:** body positivity, body neutrality, body image, adolescents, focus groups

## Abstract

Body positivity and body neutrality are increasingly referred to as alternatives to the idealized body representations on social media. These images can play a powerful role in body image development during adolescence. However, insights into how adolescents give meaning to these concepts and perceive their potential effectiveness are still limited. This study aims to fill this gap by conducting focus groups among adolescents (*N* = 27; aged between 13 and 17 years old) on (1) their conceptualization and familiarity with body positivity and neutrality, and (2) perceived effectiveness of these movements for adolescents’ healthy body image development. Moreover, we analyzed potential sex differences. The results of a thematic analysis indicated that although body positivity and body neutrality were not well-known and were only occasionally observed, most participants believed they could help, especially girls, to feel better about their bodies. The adolescents, however, were also critical in the sense that the approaches cannot be applied too excessively and that more attention is needed for body positivity for men as well.

## 1. Introduction

Idealized bodies and appearances are central on social media (Rodgers et al., 2021). As adolescents actively use social media platforms to develop their own identity and look for role models for inspiration regarding their appearance (Rodgers et al., 2021; Torres & Brito, 2022), many scholars have expressed concerns about the negative effects of exposure to this idealized content on adolescents’ body image concerns and self-esteem (Rodgers et al., 2021; Vandenbosch et al., 2022). In response, body positivity has emerged as a counter-movement on social media (Brathwaite & DeAndrea, 2022; Ladwig et al., 2024), challenging narrow beauty standards and promoting body acceptance (Cohen et al., 2021). While body positivity content has shown positive effects on body image and body satisfaction (e.g., Davies et al., 2020; Ladwig et al., 2024; Rupp & McCoy, 2023), its growth is hindered by criticisms of its continuing focus on appearance and its promotion by ideal figures (Cohen et al., 2019). Body neutrality on social media is increasingly presented as an approach that shifts the focus away from body aesthetics, emphasizing acceptance and appreciation rather than positivity or negativity toward the body (Aubrey et al., 2024; Clark, 2022). It further concerns a non-judgmental approach to the body and de-emphasizes the importance of appearance (Mulgrew & Hinz, 2024). While some scholars argue that body neutrality constitutes a conceptually distinct framework from body positivity, others highlight substantial overlap, noting that both social media movements share themes such as body functionality and self-acceptance, and that interpretations may vary across platforms and communities (Mulgrew & Hinz, 2024; Wood-Barcalow et al., 2024). Despite the potential of body neutrality, not many people are familiar with this upcoming movement (Sharp et al., 2023), which limits the knowledge on its application.

Research on body positivity and neutrality on social media has several gaps, which hinder the effective use of these techniques. Firstly, while studies on body positivity are increasing (e.g., Rodgers et al., 2023; Sanzari et al., 2023), insights on body neutrality remain limited, particularly among adolescents. Investigating this among adolescents is crucial because they are in a developmental phase characterized by physical and psychological body changes (Croll, 2005) and increased reliance on online health- and beauty-related information during this process (Park & Kwon, 2018). Moreover, adolescents are not merely passive recipients of body-related discourses but active meaning-makers who may reinterpret and negotiate these body image frameworks in ways that differ from dominant academic or media definitions. Examining their interpretations is therefore essential, not only to understand their lived experiences but also to contribute to and refine the current debate on the conceptualization of body positivity and body neutrality. Secondly, research has largely focused on female participants despite growing discussions on male body positivity and a wish for more diversity in male beauty as well (Huntington, 2023). Insights are lacking in how men perceive these concepts and whether there are differences with the female perspectives. This study addresses these gaps by using focus groups to examine adolescents’ perceptions of body positivity and neutrality, their knowledge of these movements, and their perceived impact on body image, considering sex differences.

This knowledge will be helpful for marketers who want to step away from the large focus on idealized bodies and appearances. Insights from adolescents themselves can inform best practices for addressing appearances in social media content. Moreover, influencers can benefit from these insights, informing them on how adolescents perceive their appearances and what the potential impact can be.

## 2. Literature Overview

Body image refers to individuals’ perceptions, thoughts, and feelings about their appearance (Grogan, 2021). During adolescence, young people undergo significant physical changes due to puberty, which can strongly influence their perceptions of their own bodies (Croll, 2005). These changes often lead to a highly dynamic and sometimes unstable body image. This period of physical transformation is also associated with increased sensitivity to appearance and social comparison, making adolescents particularly vulnerable to fluctuations in how they perceive their bodies (Croll, 2005). Apart from internal and psychological development processes, sociocultural theory posits that body image is also shaped by social and cultural influences, including (social) media exposure (Cafri et al., 2005). Given social media’s strong emphasis on photos and appearance, scholars have examined which body and appearance-related messages circulate online and how they affect users’ body image (Rodgers et al., 2021; Vandenbosch et al., 2022). Such appearance-related content is typically characterized by highly curated self-presentations, in which users highlight their “best selves” and often conform to narrow, culturally dominant appearance ideals (e.g., thinness, muscularity, clear skin). Such messages emphasize appearance and pressure users to attain unrealistic ideals (Maes et al., 2025; Mahon & Hevey, 2021). Social media use, and particularly interacting with such appearance-related content, has consistently been linked to negative body image outcomes in research (Rodgers et al., 2021; Siaphoo & Vahedi, 2019; Vandenbosch et al., 2022). Additionally, among adolescents specifically, the meta-analysis of Revranche et al. (2021) points to a quite consistent, although not always direct, association between exposure to appearance-related content (e.g., selfie editing; selfie posting) and a negative body image.

### 2.1. Body Positivity: Its Nature and Effects on Adolescents

The idea of body positivity was developed around the 1960s. Since the introduction of social media, and especially photo- and video-based social media, the body positivity movement has become popular as a countermovement towards the idealized appearances on social media (Cohen et al., 2021). Its popularity has been further boosted by body positivity influencers, who became role models in their aim to broaden the understanding of beauty and acceptance of different types of bodies (Cohen et al., 2021).

Body positivity challenges narrow beauty ideals by sharing images of diverse bodies and diverse body sizes to promote body appreciation and body acceptance of a wide variety of bodies and appearances (Cohen et al., 2021; Stevens & Griffiths, 2020). Body positivity content then refers to media messages that contain expressions of body self-care or body love (Cohen et al., 2021). The main themes that are being covered within body-positive content are the promotion of body love and acceptance (e.g., Harriger et al., 2023; Hallward et al., 2023; Kelly & Daneshjoo, 2019; Lazuka et al., 2020), inner positivity (e.g., Lang et al., 2023; Lazuka et al., 2020), and body functionality (Lazuka et al., 2020). Body functionality within body positivity refers to the appreciation for what your body is capable of, which is more important than aesthetics (Lazuka et al., 2020).

Body-positive content on social media appears in both textual and visual forms. Textually, it includes captions and hashtags promoting body acceptance and diverse notions of beauty (e.g., #bodypositivity, #bopo) (Cohen et al., 2019; Harriger et al., 2023). Visually, it features diverse body types and underrepresented bodily characteristics, often through unedited images (Cohen et al., 2021; Lazuka et al., 2020; Lang et al., 2023).

Several studies pointed to positive effects of body-positive content on women’s body satisfaction, appearance concerns, body appreciation, and positive feelings, both in experimentally controlled settings as well as natural environments (e.g., Davies et al., 2020; Ladwig et al., 2024; Rupp & McCoy, 2023; Stevens & Griffiths, 2020; Vandenbosch et al., 2022). A small but growing body of research has examined the role of body-positive content in adolescents’ body image specifically; for example, Kvardova et al. (2022) found that adolescents who intentionally seek out body-positive content report a more positive body image. Trekels and Eggermont (2024) showed that exposure to idealized appearance content is positively related to adolescents’ body dissatisfaction, whereas exposure to body-positive content is negatively related to body dissatisfaction. However, the evidence remains mixed; for instance, no within-person associations were found between exposure to body-positive content and appearance-related prosocial tendencies among adolescents (Kvardova et al., 2025), and Vranken et al. (2025) reported positive associations between exposure to body-positive content and negative body image components, including self-objectification, internalization of appearance ideals, and appearance comparisons with peers. These inconsistent findings call for a deeper discussion of the psychological mechanisms involved.

Several studies explain the effects of body-positive content using social comparison theory (Festinger, 1954), which posits that individuals evaluate themselves through upward and downward comparisons. Exposure to idealized content on social media typically triggers negative upward comparisons, leading to poorer body image among adolescents (De Vries & Kühne, 2015; Vandenbosch et al., 2022). On social media, exposure to idealized appearance content can evoke negative upward social comparison processes (Vandenbosch et al., 2022), meaning that adolescents perceive the people in such posts as superior and unattainable comparison targets (i.e., they have appearances that seem unreachable for themselves), resulting in a negative body image (De Vries & Kühne, 2015). Yet, in the case of body positivity, upward comparisons may function differently. Building on the selective accessibility model (Mussweiler, 2003) as outlined by de Lenne et al. (2023a), body-positive posts often feature non-idealized models who resemble the average person, increasing perceived similarity and fostering assimilative comparison processes. Although these models may still be perceived as upward comparison targets, for instance, because they display confidence, body acceptance, or rejection of narrow appearance ideals, such assimilative upward comparisons are assumed to be more attainable and can inspire or reassure adolescents, thereby contributing to more positive body image outcomes (de Lenne et al., 2023a). Rodgers et al. (2023) indeed found that exposure to body-positive individuals on social media helps adolescents to distance themselves from narrow appearance ideals. Simultaneously, body positivity content can have positive effects on body image through downward social comparisons. In this case, body positivity influencers may be perceived as inferior comparison targets as they deviate from the internalized appearance ideals (de Lenne, 2022). Contrasting one’s appearance with comparison targets who are perceived as inferior, in turn, results in greater satisfaction with one’s own body and appearance.

However, there also circulate concerns about the potential drawbacks of the body positivity movement (Cohen et al., 2021) due to its continuing focus on appearance (Webb et al., 2017). Although body positivity claims not to be about the perfect body, the body is still central. In more than one-third of the body-positive posts on Instagram, there is limited and sexualizing clothing, meaning that the body is central in the picture (Cohen et al., 2019; Lazuka et al., 2020). One study found that body-positive images with a strong focus on appearance promoted self-objectification to a similar extent as thin body images (Cohen et al., 2021; Vandenbosch et al., 2022), which can reinforce the preoccupation with the body.

Moreover, body-positive messages are oftentimes spread by skinny and traditionally beautiful figures, which signals an authenticity problem (Brathwaite & DeAndrea, 2022). TikTok is considered especially problematic in this regard, as many videos with the tag body positivity were spread in sexually objectifying poses by figures with bodies that comply with sociocultural appearance ideals (Hallward et al., 2023), which can create cognitive dissonance among the audience (Hallward et al., 2023).

### 2.2. Body Neutrality

Given the criticism of body positivity, attention is increasing to another approach; that is, body neutrality (Cohen et al., 2021; Cowles, 2022), a concept that has been introduced by body image coach Anne Poitier as an alternative to body positivity (Smith et al., 2023). Analyzing different definitions and conceptualizations of the term on popular websites, Pellizzer and Wade (2023) concluded that three elements are central when defining body neutrality: “Our feelings about our body change constantly so are best mindfully observed without judgment; a central focus on what our body allows us to do and appreciating this will lead us to respect and care for our body; and acknowledgment that our self-worth encompasses both intrinsic qualities and extrinsic passions and deemphasizes our appearance” (p. 439). Body neutrality is often seen as the middle ground between loving and hating your body, promoting neither positivity nor negativity towards the body, but acceptance and respect for what it is and what it is capable of (Clark, 2022). Recent scholarship has noted that body neutrality is conceptually fluid and still evolving as a media-driven concept (Wood-Barcalow et al., 2024). These authors note significant overlap with body positivity, particularly around the appreciation of body functionality. However, other scholars claim that body neutrality should be articulated as a related yet conceptually distinct framework; one that prioritizes non-appearance-related values, centers everyday bodily functionality, and encourages emotional detachment from appearance-based self-evaluation (Mulgrew & Hinz, 2024). Ladwig et al. (2024) bring together the different perspectives in the idea that both movements focus on aspects of positive body image, but in a different way: in body neutrality, the focus is on helping individuals value their body’s functions whereas, in body positivity, the focus is on loving and accepting diverse bodies, including your own, body’s appearance. This work follows this conceptualization, recognizing the importance of the relatedness and at the same time uniqueness between both concepts.

Regardless of its conceptualization, the body neutrality approach has become increasingly popular in the media since the mid-2010s (Cowles, 2022). One common form of expression is by focusing on body functionality instead of appearance, meaning that you see the body as something functional for which you do certain things, but nothing more than that (Clark, 2022). As explained above, body functionality is also a core construct of body positivity, which introduces some ambiguity in the literature due to the differing perspectives of these concepts. Another way to express body neutrality is by seeing your self-concept as completely based on your personality, intelligence, and social environment and thus disconnected from your body (Clark, 2022). Expressions of #BodyNeutrality have regularly been observed on TikTok, with most of these messages being informational on what body neutrality is (Hallward et al., 2023). Body-neutrality themes detected in the content analysis of Aubrey et al. (2024) on TikTok were Size Inclusivity (i.e., people’s activities/preferences/words should not be based on body size), Adaptive Self-Investment (i.e., self-care focused on health), Body Appreciation (i.e., respect for what one’s body can do), and No Judgment (i.e., a person’s worth should not be based on appearance). This content is very popular, with more than 700.2 million views for #BodyNeutrality content (Hallward et al., 2023). On TikTok, this number rises to over one billion (Mancin et al., 2024).

Body functionality, which has been incorporated in both the body neutrality (e.g., Smith et al., 2023) and body positivity movements (e.g., Lazuka et al., 2020), has increasingly been the subject of intervention and effect studies. Emphasizing the functional appreciation of the body has also been related to positive body outcomes such as higher body satisfaction (e.g., Ladwig et al., 2024), a better mood, and improved body image (e.g., Mulgrew & Courtney, 2022). A growing number of studies examine how functionality-oriented messaging specifically operates on social media. Mancin et al. (2024) reported that functionality was the dominant theme in #bodyneutrality videos. Experimental evidence further supports the potential of body-neutral content. Brathwaite et al. (2023) showed that posts perceived as body-neutral were judged more morally appropriate, which in turn promoted inclusive beauty standards. Seekis and Lawrence (2023) found that brief exposure to body-neutral TikTok content—compared to thin-ideal or control content—led to greater functionality appreciation, higher body satisfaction, more positive mood, and fewer upward appearance comparisons.

In conclusion, in light of the discussion on the positioning of body functionality in relation to body positivity versus body neutrality, we tend to follow the prior literature, conceptualizing body neutrality primarily as an approach that emphasizes body functionality and de-emphasizes appearance-based evaluation, whereas body positivity focuses more explicitly on acceptance and appreciation of diverse body appearances (Mulgrew & Hinz, 2024; Ladwig et al., 2024).

### 2.3. This Study

The findings above suggest that body neutrality has great potential for empowering social media users in their body image, yet research on this concept among adolescents is limited. The recent work of Rodgers et al. (2023) and Maes et al. (2025) provides first insights into young people’s responses to body-positive social media content, particularly highlighting processes like broadening beauty standards and resisting appearance ideals. Previous research, however, started from pre-defined conceptualizations. Although we, as researchers, conceptualize body positivity and body neutrality as distinct yet related concepts—where body positivity emphasizes acceptance and appreciation of diverse body appearances, and body neutrality focuses on functionality and reducing the centrality of appearance (Mulgrew & Hinz, 2024; Ladwig et al., 2024)—adolescents may conceptualize and experience these movements differently. Our study builds on existing research by examining adolescents’ own conceptualizations of these concepts, their familiarity with them, and perceptions of effectiveness, and by exploring how these movements are understood in relation to healthy body image development across sex.

Such insights are needed because a consistent tension appears in the literature: although #bodypositivity content is widely documented in content analyses (Aubrey et al., 2024; Clark, 2022; Cohen et al., 2019), several studies show limited awareness on the audience side. Women in the study of Ando et al. (2021) rarely encountered such posts and struggled to name role models; similar patterns emerged among students and healthcare professionals (Langnes & Walseth, 2023; Sharp et al., 2023). Sanzari et al. (2023) further found that only a minority of adolescents actively seek out this content. While studies such as Rodgers et al. (2023) and Maes et al. (2025) examine adolescents’ general responses to body-positive posts, they do not address adolescents’ spontaneous interpretations of everyday feed encounters or their reflections on applicability and gender inclusivity.

For body neutrality, unfamiliarity is an even greater barrier: Sharp et al. (2023) showed that most healthcare professionals had never heard of the concept, though they saw it as more promising once explained. Evidence on adolescents’ understanding of body neutrality is almost entirely missing.

Taken together, there is a need for more qualitative research that examines how adolescents themselves conceptualize body-positive and body-neutral movements, how they distinguish between them, and why they perceive such messages as potentially helpful. Qualitative focus groups are particularly suited for this purpose, as they provide adolescents with a context to express and negotiate their own perspectives, reflect on real-world social media practices, and uncover tacit beliefs, misunderstandings, and interpretive processes.

The present study, therefore, aims to fill in a research gap by conducting a focus group study among adolescents, aiming to answer how they conceptualize, perceive and experience both body image movements on social media. More specifically, we split this research question into two subquestions:

RQ1: How do adolescents conceptualize, perceive and experience body positivity and body neutrality on social media?

- How familiar are adolescents with these movements?
- How do adolescents judge the perceived effectiveness of these movements in improving adolescents’ body image?

### 2.4. Sex Differences in Perceptions of Body Positivity/Neutrality

Almost all research on the effects of body-positive and body-neutral content has focused on women or adolescent girls. This aligns with the idea that girls, in general, are more focused on their bodies, consume more appearance-based media (Marengo et al., 2018) and struggle more often with body concerns and self-objectification (Salomon & Brown, 2019). Content analyses also indicated that body-positive messages are mostly focused on women (Cohen et al., 2019) and are more searched for by women (Sanzari et al., 2023). This difference is also observable in adolescents’ own body-positive posting behavior. Comparing the Facebook posts of adolescent boys and girls, Torres and Brito (2022) stated that boys put more emphasis on body appreciation and functionality by posting about sports, whereas girls highlight appearance. Looking at the effects, however, it appeared that only women decreased in body satisfaction after exposure to idealized body ideals, yet negative vs. positive effects occurred for both women and men when participants were specifically asked how viewing the idealized Instagram posts vs. the body positivity posts made them feel about certain body parts (Pritchard & Button, 2023).

Despite the large focus on body positivity among women, recent trends in the body positivity movement for men can be observed, and the idea grows that women and men have an equal need for body positivity (Clay & Brickell, 2022). Similarly to women, there are strong standards on what an ideal male body should look like, characterized by a mesomorphic body shape with defined muscles (Huntington, 2023). However, research also pointed to increasing levels of body dissatisfaction among men (Dakanalis et al., 2015) and muscle dysmorphia (i.e., increased concerns about being insufficiently muscular) due to this kind of content (Tod et al., 2016). As a result, there is an increasing wish for a more inclusive vision of what masculinity is and what a masculine body should look like (Huntington, 2023). The idea of male body positivity is that different types of body sizes and shapes should be considered masculine.

A couple of studies tested the effects of plus-size models and diverse models in the advertising context on men and found positive effects on men’s feelings of physical attractiveness, muscle satisfaction, and overall mood (e.g., Agliata & Tantleff-Dunn, 2004; Hargreaves & Tiggemann, 2009). Comparing muscular, slim, plus-size, and overall diverse models, the study of de Lenne et al. (2023b) found that both diverse and plus-size models in ads generated positive effects on men’s body image, but not on other well-being variables. Compared with women, it has been argued that men might be less attentive to the bodies that are being shown in advertising (de Lenne et al., 2023b). This finding has been interpreted through the idea of hegemonic masculinity (Connell, 1998), which emphasizes men’s adherence to norms such as emotional restraint, self-reliance, and the rejection of behaviors associated with femininity. Following this reasoning, it could be that men are reluctant to express their acceptance of non-idealized bodies as this contradicts the hegemonic masculinity narrative, and might thus be seen as a reflection of weakness or femaleness (Hargreaves & Tiggemann, 2009).

Given these recent developments, it seems interesting to determine whether there are sex differences in adolescents’ conceptualizations, perceptions, and opinions about both body image frameworks:

RQ2: Are there differences between boys and girls in their perceptions of and experiences with body positivity and body neutrality?

## 3. Methods

### 3.1. Procedure

To answer our research questions and subquestions, we conducted focus groups, as this method allows us to gather in-depth insights on this “new” issue and to capture potential dynamics among adolescents, as opinions and feelings about the body are largely influenced by peer conversations (Kenny et al., 2017). Participants were approached via several secondary schools in the region of Flanders. A total of 27 adolescents participated in the focus groups, divided across five groups. The classes that participated were selected by the school direction team, depending on availability. All participants were Belgian and aged between 13 and 17 years old. They were evenly divided across both sexes (14 girls and 13 boys). See Table 1 for an overview of the participants within the focus groups. Focus groups were organized according to sex (no mixed groups) and age (no more than two years difference) and consisted of a maximum of seven participants. Meta-analytic research analyzing how many focus groups are necessary for studying health-related topics suggested that for homogeneous samples like ours and simple topics focusing on health opinions, experiences and cultural norms, a total of three to six focus groups should be enough to capture 90% of the themes and experiences (Guest et al., 2017).

The focus groups had a semi-structured approach and were combined with prompts. All focus groups were conducted by one researcher, audio-recorded, and transcribed verbatim. The semi-structured focus groups covered predetermined topics like body positivity and body neutrality, but allowed room for participant input. The conversations were organized at school and consisted of three parts: an introduction on social media use and body representation as a conversation opener, followed by discussions on body positivity and body neutrality. For the discussions on body positivity and body neutrality, we started with the question of how participants would define these concepts and whether they could provide examples. In that way, we follow their interpretations and distinguish ourselves from previous studies. To reduce the risk of shaping participants’ views, definitions were given only when necessary—for instance, when adolescents were unable to articulate a working definition. This is a limitation, but it was necessary because when the understanding of the concepts was too low, it was not possible for the participants to reflect on the perceived effectiveness. For body positivity, all groups came to an understanding that was sufficient to reflect on effectiveness, but for body neutrality, that was not the case in groups 3, 4 and 5. In these instances, the researcher explained the concepts based on the conceptualizations and characteristics that were mentioned during earlier focus groups and not based on our academic definitions, to stick with adolescents’ wordings (e.g., “body neutrality is not about loving your body or hating your body, but that the body is simply a part of you.”). Such clarifications were explicitly framed as “other groups defined it as” and were introduced after participants’ initial interpretations had been documented. In that way, we aim to avoid priming effects while still ensuring conceptual clarity for the remainder of the discussion.

Photo prompts were used during the conversations. The prompts were balanced between female and male body-positive content. We selected a diversity of prompts, with some bodies ranging from small, but realistic, to normal to large. By only introducing the prompts after participants had expressed their initial ideas, we aimed to minimize the extent to which example stimuli might influence participants’ interpretations of the movements. The prompts thus served to deepen the conversation rather than to direct it.

The interview guide, including the selected prompts, was pre-tested with four adolescents to ensure comfort, question clarity, and prompt effectiveness, with minor adjustments made afterward. Ethical approval was obtained from the Ethics Committee for the Social Sciences and Humanities of the University of Antwerp. All participants signed an informed consent at the start of the focus group. In accordance with the guidelines of the Ethical Commission of the University of Antwerp for research involving adolescents aged 13 years or older, parents were informed via an information letter.

After five focus groups, data saturation was considered to be reached. Throughout the coding process, we continuously compared codes and themes across focus groups. In the last focus group, no substantially new codes emerged. The findings from that group confirmed the existing thematic patterns and discussion on the conceptualizations. Additionally, for instance, the finding that body positivity and body neutrality might be helpful, but cannot be too extreme, emerged as in the previous focus groups. While the notion of saturation is debated within reflexive thematic analysis (see, e.g., Braun & Clarke, 2021), we use it here pragmatically to indicate the point at which further data collection no longer yielded meaningfully new thematic insights relevant to our research questions.

### 3.2. Data Analysis

We adopted a reflexive thematic analysis of the focus group data, informed by constructivist grounded theory (Corbin & Strauss, 1990). Coding followed a multi-stage process. Initial open coding identified meaningful segments (e.g., “no photoshop,” “self-confidence”), which were iteratively refined. The codes we used during this first stage were descriptive in nature, aimed to stay close to participants’ own language to conceptualize the concepts, following a participant-centered approach.

Axial coding grouped these descriptive codes into broader thematic and categories: Conceptualization, Familiarity, Examples, Effectiveness, and gendered interpretations and subcategories. When participants explicitly referred to sex differences (e.g., describing body image concerns as more relevant for girls or as a taboo among boys), these paragraphs were coded as part of their expressed understandings, clustered in the category ‘gendered interpretations’. An overview of the descriptive codes and thematic categories can be found in Table 2.

Next, in the analytical stage, we adopted a more deductive, question-driven approach to address our research questions. Firstly, we compared emergent themes to existing academic definitions and conceptualizations of both body image frameworks to answer RQ1. In addition, we conducted a comparative reading across focus groups consisting of boys and girls to address RQ2. This involved side-by-side examination of coded segments to identify recurring similarities and differences in how adolescents discussed and interpreted body image frameworks.

NVivo was used to facilitate data organization and iterative coding. An overview of the codes can be found in Table 2. Intercoder reliability was assessed on 30% of transcripts (Cohen’s κ = 0.79), with discrepancies resolved through discussion. This approach ensures rigor, transparency, and grounded interpretation of adolescents’ perspectives.

## 4. Results

The analysis yielded several themes that structured adolescents’ understandings and experiences of body positivity and body neutrality. Across focus groups, participants described: (1) limited conceptual understanding, particularly regarding body neutrality; (2) reliance on recognizable content formats, such as “Instagram versus reality” posts, as interpretive anchors; (3) gendered patterns of recognition and engagement, with boys and girls differing in how they relate these movements to their own experiences; (4) perceived benefits and emotional relief, mainly among girls, in response to non-idealized portrayals; and (5) concerns about potential extremity and unintended consequences in relation to body positivity. These themes are elaborated below within the structure of RQ1a (knowledge and familiarity) and RQ1b (perceived effectiveness), while attending to differences across sex (RQ2).

### 4.1. Knowledge and Familiarity with Body Positivity and Body Neutrality

Overall, adolescents were not very familiar with both body image frameworks, with familiarity with body neutrality varying more strongly across focus groups than familiarity with body positivity. To assess their knowledge and conceptualizations (RQ1a), we first asked them to define the concepts and provide examples. When discussing body positivity, adolescents mainly referred to influencers who share “real” photos without filters or Photoshop. They described it as showing features that cannot be changed, such as stretch marks or scars. Some girls additionally mentioned plus-size models as examples.

“I think it is about accepting how you see yourself and that everything is ok and that there are fat people and skinny people. But you are who you are and sometimes there are people who are fat, but there is nothing they can do about that, they are born like that, so you have to accept that and be happy and not always compare yourself with everyone.”

(Focus group 5, girls; 13–14 years old)

In one group, the girls started talking about Instagram versus real posts. As this appeared to be something all adolescents were more familiar with, we also included this topic in the focus groups that followed. The Instagram versus real post concerns a post consisting of a combination of two times the same picture, one time without Photoshop or filters and one time the idealized version. Many adolescents like this idea as it allows influencers to show their best selves, as this is what social media is all about, but at the same time, the audience can observe and learn that what they see is not always real.

“These guys they always post pictures with the right light, immediately after a workout, then you have good blood flow. But in the morning when they wake up, it is not like that. I think it is crazy, when you see these pictures and videos, you think wow. But then you see the real pictures and you realize that they actually have a similar body as you have.”

(Focus group 4, boys; 16–17 years old)

“They can show: this [non-idealized pictures] is also an option, but I prefer it like this. And then the audience will know and they will see, yet it does not matter how I look. You can show that with the two pictures”.

(Focus group 2, girls; 15–16 years old)

When discussing body positivity, some adolescents referred to well-known examples such as Lizzo (@Lizzobeeating), Joann van den Herik (@joannvdherit), and Rianne Meijer (@Rianne.meijer), who are known for body positivity or Instagram-versus-reality posts. Remarkably, in the boys’ focus groups and in one of the girls’ groups, none of the participants could name any body-positive influencers, even though they could easily list influencers with highly idealized bodies. Participants in these groups reported seeing body-positive content mainly on YouTube and TikTok, which they described as platforms that show everyday life and routines, in contrast to Instagram, which they perceived as focused on idealized images.

“It is changing. On TikTok for instance, you have it very often, but also on Instagram Reels, body positivity and stuff like that. When you see people, such as Spencer Barbosa, she also does it a lot. So yes, there is more diversity.”

(Focus group 5, girls; 13–14 years old)

Whereas in all focus groups, there was some knowledge on body positivity, which was enough to develop a working definition within their group, that was less the case for body neutrality. Some adolescents in these discussions linked it to the idea that social media should not be about bodies at all, but more about being yourself. In one group, the concepts of body-positivity and body-neutrality were messed up. As the latter quote shows, the girls tried to explain body positivity but actually referred to the main idea of body neutrality.

“It is Instagram, not everything needs to be about bodies. It should be about what you are doing, I don’t think you should constantly put your body on it. That is not the purpose of Instagram.”

(Focus group 1, girls; 16–17 years old)

In the three groups that were unable to develop their own working definition of body neutrality, the researcher gave them a description based on the previous focus groups to continue with during the conversation (e.g., “body neutrality is not about loving your body or hating your body, but that the body is simply a part of you.”). As a result, participants’ engagement with body neutrality was not fully equivalent across groups, as some responses were based on spontaneously articulated understandings, whereas others were prompted by the researcher’s explanation. Interestingly, in the one focus group, after giving our explanation of the concept, participants immediately also linked it to inner beauty:

“I agree, because I also think that we should not always look at the body, you also have to look at the personality.”

(Focus group 5, girls, 13–14 years old)

### 4.2. Perceived Effectiveness of Body Positivity and Body Neutrality on Adolescents’ Body Image

After discussing the meaning of body positivity, adolescents discussed the perceived effectiveness in improving their body image (RQ1b). The variation in how the concept was introduced was taken into account here. In several groups, especially girls’ groups, there was some level of agreement that these pictures can be helpful to feel better about one’s body. The issue of comparing yourself with others was mentioned by several girls and echoed by others.

“I easily compare myself with others. And then I like this better. When I see very skinny models on Instagram, then yes I compare myself. I feel bad about it and it puts me in a negative mood. But with the plus size models, I do not have that; they give me a more positive feeling.”

(Focus group 1, girls; 16–17 years old)

“I think many girls still feel bad about how they look and I think that if you would see this kind of content more often on Instagram, that can help. For boys as well maybe!.”

(Focus group 1, girls; 16–17 years old)

In the boys’ groups, however, there was some variation on this opinion. As mentioned above, several boys indicated that feeling bad because of idealized bodies on social media was not a salient issue among them, which also makes body positivity for men be perceived as less necessary.

“I think it is more problematic among girls than among boys; they look up to these perfect bodies. So, I think for girls it is good that these ideas of body positivity exist; that they know that their bodies are also normal, that it does not need to be perfect.”

(Focus group 3, boys; 14–15 years old)

“I don’t think body positivity exists for men. I also don’t think that many men struggle with body positivity, because they just more easily accept themselves.”

(Focus group 3, boys; 14–15 years old)

Remarkably, both boys and girls identified perceived limitations to the body positivity movement. Adolescents in several groups expressed concerns about the possibility of body positivity being taken to an extreme. Specifically, adolescents in these groups worried that an overemphasis on body positivity might inadvertently promote unhealthy behaviors by suggesting that all body conditions are acceptable, even when they may not support overall health and well-being.

“I think it is good as long as it is not too extreme.”

“Yes, if you say such a body is better than a trained body, so you don’t need to go to the gym, you can just do nothing; that is not what you want.”

“Yes, or sometimes people say that people who go to the gym are against body positivity; that is fit shaming. Then it goes too far.”

(Focus group 3, boys; 14–15 years old)

“I think it is good to a certain level. I mean if people are obese, that is just not healthy. I mean I don’t see the point in supporting that. Ok you can be a bit fatter, and if you are happy with that, that is perfect. But if it becomes unhealthy, then you can have the opposite effect. That is the same when you are super super skinny, like anorexia, which is also unhealthy and should not be promoted. You need to help the people.”

(Focus group 4, boys; 16–17 years old)

The latter quote makes a nice bridge to the potential of body neutrality as it implies it should not be about what is good or bad.

“I don’t think this kind of picture [body-positive pictures] is better or worse than someone posting the same pictures with a skinny posture. A body is a body, right?”

(Focus group 1, girls; 16–17 years old)

Adolescents generally expressed positive evaluations of body neutrality, often more favorable than those expressed toward body positivity, although this comparison should be interpreted with caution given the differences in familiarity and the fact that body neutrality was, in some cases, introduced by the researcher. After the concept and examples were discussed, most participants considered body neutrality a valuable—perhaps even preferable—approach, though they noted it is rarely encountered. Notably, positive evaluations of body neutrality emerged across groups, including those in which the concept had to be introduced, suggesting that its underlying principles may resonate with adolescents even when they are not part of their existing vocabulary.

Within the boys’ focus groups, a more limited engagement with body image frameworks was observed compared to the girls’ groups. However, this does not imply uniformity in boys’ perspectives. One focus group predominantly framed body positivity and the broader issue of body image as less relevant for men, arguing that boys are less affected by body image concerns. Although this vision was put forward by two boys, the others seemed to agree by nodding and confirming. In contrast, the other boys’ group included more reflective positions, with several participants explicitly acknowledging that boys may also experience limited diversity in body ideals and reduced social support for body-related concerns. This becomes clear within the following discussion among several boys in this group:

“It is just less a thing among men. It is more like you train to have a nice body, and that nice body is for almost everyone the same. They have the same goal. Whereas body positivity among women means that different types of bodies are being accepted. That is just not a thing about men.”

(Focus group 3, boys; 14–15 years old)

R1: “All boys just want the same kind of body; whereas for girls it is way more normal that they have different kinds of bodies.”
R2: “Body positivity is something only for women. We already said it earlier, for men there only seems to be one ideal body, but for women that is not the case, there are several ideal bodies.”
R3: “It seems like we have a new ideal, I mean that all bodies are being accepted among women and girls as well. And that makes them, you know, they feel better about their bodies, but it is like they say, for boys nobody will say about a men “Hé you have some overweight, but that is also perfectly beautiful. No that is not how our society thinks right now.”
R4: “I think in general men are less supported in that, in the body positivity.” (Focus group 4, boys; 16–17 years old).

Overall, these findings suggest gendered differences not only in the content of adolescents’ interpretations of both body image frameworks, but also in the breadth and elaboration of these interpretations.

## 5. Discussion

The present study explored adolescents’ conceptualizations and experiences with body positivity and body neutrality on social media, as well as their perceptions of how useful these movements are in improving positive body image.

### 5.1. Familiarity and Conceptualization with Body Positivity and Body Neutrality

Firstly, our focus groups revealed that most adolescents are familiar with body positivity but not with body neutrality, and that familiarity with body neutrality differed between groups. They conceptualized body positivity as showing “real” images that challenge societal beauty ideals, such as scars or plus-size bodies. Some linked it to being yourself on social media, aligning with body neutrality, though they did not recognize the term. Importantly, this variation in familiarity was also reflected in the way the concept of body neutrality entered the discussions. In some focus groups, participants were able to describe elements of body neutrality spontaneously, whereas in others, the concept had to be introduced by the interviewer. The wording that the interviewer used was based on previous focus groups, and not on an academic definition, however, evaluations of body neutrality should still be interpreted with caution, as they were, in some cases, not entirely spontaneous understandings.

The academic discussion on whether body functionality fits under body positivity or body neutrality was not reflected in the conversations, as our participants did not spontaneously link body functionality with the movements. The fact that adolescents in one focus group even mixed up the concepts of body positivity and body neutrality links nicely to the academic discussion on whether these are two distinct movements or rather two different expressions of the same idea (e.g., Wood-Barcalow et al., 2024; Mulgrew & Hinz, 2024). Adolescents’ difficulty in distinguishing between the body image movements suggests that the conceptual separation that exists in academic theorization (Mulgrew & Hinz, 2024; Ladwig et al., 2024) is not mirrored in everyday social media practice. In contrast to our academic conceptualization distinguishing between body positivity and body neutrality, adolescents appeared to categorize a wide range of non-idealized and authentic body portrayals under the umbrella of “body positivity,” regardless of whether the content reflected positivity or neutrality. Moreover, adolescents’ unfamiliarity with body neutrality—despite recognizing some of its themes—suggests that academic definitions may not fully align with how these concepts circulate among younger audiences and are made meaningful in youth social media cultures. Part of the explanation, however, might also have to do with the fact that adolescents rarely encounter such content. Several adolescents noted that body positivity content is only a small part of their social media diet, with idealized appearance content still dominating—a pattern supported by existing research (Sanzari et al., 2023).

The central contribution of this study, therefore, lies in demonstrating that adolescents do not clearly distinguish between body positivity and body neutrality, but instead understand a wide range of non-idealized content under the broad label of “body positivity”, as reflected in their own language and interpretations rather than in academic definitions. By foregrounding adolescents’ own interpretations of body positivity, our findings further extend existing predominantly adult-focused research by demonstrating that conceptual interpretations may vary by age group. For body neutrality conceptualizations, this variation is also present within the age group, given the varying levels of familiarity with this concept.

### 5.2. Perceived Effectiveness in Improving Positive Body Image

Almost all participants recognized the potential benefits of body positivity content, aligning with research showing that posts promoting body appreciation and resisting appearance ideals are seen as helpful (Rodgers et al., 2023). However, several of them remained skeptical, believing that idealized appearance content dominates social media and triggers harmful upward comparisons (De Vries & Kühne, 2015; Vandenbosch et al., 2022). This aligns with concerns about body positivity’s focus on appearance and objectification (Webb et al., 2017). Body positivity may thus be perceived by adolescents as a positive shift, yet to actually apply its principles to one’s own body may still be challenging (de Lenne, 2022).

Adolescents in this study generally expressed more favorable evaluations of body neutrality content compared to body positivity. However, this finding should be interpreted taking the differences in familiarity across groups into account. Interestingly, the more favorable evaluation of body neutrality was found across focus groups, including those in which the concept had to be explained by the researcher. This suggests a certain consistency in how adolescents respond to the underlying principles of body neutrality once these are made explicit. Moreover, it suggests that the explanation given by the researcher aligns with the conceptualizations spontaneously constructed in the other focus groups. At the same time, these responses on effectiveness should not be interpreted as reflecting a fully pre-existing preference. Rather, they indicate that the concept of body neutrality may be perceived as appealing when presented and explained.

The effectiveness of body neutrality was described in contrast to the perceived limitations of body positivity. Most participants described body positivity as potentially becoming “too extreme” by inadvertently encouraging unhealthy behaviors, which may explain why body neutrality was viewed as a more acceptable alternative. Another possible explanation for why adolescents seemed to favor the principles of body neutrality is that it shifts attention away from appearance evaluation altogether, thereby reducing pressure to actively love or celebrate one’s body (Mulgrew & Hinz, 2024). Rather than demanding a positive self-evaluation, body neutrality may allow adolescents to relate to their bodies in a more pragmatic way, which could be particularly appealing during a developmental phase characterized by heightened self-consciousness and peer comparison (Croll, 2005). Yet, adolescents did not articulate such underlying mechanisms explicitly. This may reflect the constraints of focus group discussions, where peer norms and social desirability may limit deeper reflection on personal experiences (Hollander, 2004).

Across focus groups, regardless of their initial familiarity with the concepts, some adolescents perceived the effectiveness of both body image frameworks as limited because they rarely encounter such content spontaneously. They noted that idealized appearances still dominate their feeds, especially on Instagram—despite research identifying the platform as a hub for body positivity (Stevens & Griffiths, 2020). This mismatch between scholarly expectations and adolescents’ lived experiences has implications. It underscores the need for research on the algorithmic visibility of such content across age groups, and it suggests that interventions may need to challenge dominant appearance norms more directly rather than merely adding body-positive or neutral content to the platform.

### 5.3. Sex Differences in Experiences with Body Positivity and Body Neutrality

Most boys and girls were similarly familiar with both body image frameworks, but differed in perceived usefulness. The majority of the girls considered such content helpful to counter idealized appearances, whereas one boy’s focus group viewed body positivity as unnecessary, framing the negative effects of idealized content primarily as a girls’ issue. This aligns with prior research showing that boys perceive diverse body representations on social media as less helpful (Rodgers et al., 2023). Our findings suggest a third-person effect: most boys perceived body positivity as effective for girls but not for themselves. This is consistent with evidence that boys and men tend to downplay the personal impact of idealized content and body comparisons (Maes et al., 2025; Jankowski et al., 2018). Earlier focus group research similarly showed that boys deny media influence on body image and associate body image concerns with femininity or homosexuality (Hargreaves & Tiggemann, 2009). This third-person effect may reflect broader gender socialization and hegemonic masculinity norms, which encourage emotional invulnerability and self-reliance, leading boys to minimize their own vulnerability and attribute it to others (Exner-Cortens et al., 2021; de Lenne, 2022).

Nonetheless, some boys and girls did acknowledge the need for more attention for men in body positivity. This corroborates with the more universal criticism that has been out on the body positivity movement in that it should enlarge its scope to include greater diversity of sex and age, amongst others (Cohen et al., 2021). Additionally, the few empirical studies that have been conducted on male body positivity focused on adults (e.g., de Lenne et al., 2023b). There thus seems to be a need to stimulate male body positivity among adolescents and unravel its positive implications for boys’ body image.

### 5.4. Limitations

Several limitations should be noted. First, the focus groups consisted of adolescent peer groups, which may have amplified the observed third-person effect. Adolescents’ heightened sensitivity to peer evaluation (Kenny et al., 2017) may have made it difficult for them to discuss personal negative effects. Future research could explore these issues through in-depth interviews.

Second, the study was conducted in Belgium, a Westernized context that has been disproportionately represented in body image research (Landor et al., 2024). Further research should examine how adolescents with racialized and minoritized bodies experience body positivity and neutrality, and how these movements are perceived in terms of inclusivity and intersectionality. Moreover, participants were from the same school, which further limits the generalizability of the findings. Depending on the school culture and specific topics that are covered during courses, it could be that our participants’ experiences and conceptualizations are different from those of other students. Future research should therefore recruit from different schools in different regions.

Third, larger and more diverse samples—particularly including more adolescent boys and participants from different schools—would enhance generalizability. Future studies should also consider background characteristics such as ethnicity, sexual orientation, and gender identity, rather than focusing solely on sex.

### 5.5. Practical Implications

While the present study is exploratory and based on a relatively small, context-specific sample, the findings suggest several tentative implications that may be relevant for future research and practice.

For marketers interested in responsible communication with adolescents, the findings point to the potential value of further exploring approaches that align with body neutral principles within social media campaigns given that adolescents appeared to respond positively to such ideas when they were introduced and explained.

Importantly, adolescents in this study did not consistently differentiate between body positivity and body neutrality in the ways these concepts are defined in the academic literature. This suggests that any such approaches may be most effective when they do not rely on explicit academic terminology, but rather use language and representations that resonate with adolescents’ everyday understandings of bodies and appearance. For example, reducing visual and evaluative emphasis on appearance and highlighting functional, diverse, and non-idealized bodies may reflect body neutral ideas without requiring adolescents to be familiar with the concept itself, or to have previously engaged with it in their everyday social media use. Future experimental research could examine whether and how such framings in influencer marketing relate to adolescents’ body image.

For educators, parents, and counselors, the findings indicate that adolescents report rarely encountering body positive or body neutral content organically in their social media environments. This suggests that educational efforts related to intentional feed construction, such as critically discussing algorithms or introducing accounts that promote diverse bodies and holistic understandings of well-being, may be worth exploring further. Educational contexts may also provide a space to gradually introduce and critically discuss concepts such as body neutrality, helping adolescents to develop a shared language for reflecting on body ideals and appearance norms.

Finally, at a broader level, the findings highlight the continued dominance of appearance ideal content in adolescents’ social media environments. For policymakers and advocacy groups, this underscores the relevance of further examining how platform design, recommendation systems, or policy initiatives might increase the visibility of more diverse and health-promoting representations.

## Figures and Tables

**Table 1:** Overview of the focus groups.

	Number of Participants	Sex of the Participants	Age
Focus group 1	4	Female	16–17 years old
Focus group 2	4	Female	15–16 years old
Focus group 3	7	Male	14–15 years old
Focus group 4	6	Male	16–17 years old
Focus group 5	6	Female	13–14 years old

**Table 2:** Overview of the codes.

Conceptualization	Body positivity	No manipulation
		No photoshop
		Acceptation
		Realitstic
		Expression of self Confidence
	Body neutrality	Embracing yourself
		Being yourself
		More than 1 ideal
		Behind the scenes idee
Familiarity	Large focus on bodies on social media	
	Fitness context	
	Not spontaneous	
	Influencers	
	Not actively looking for it	
Examples	Plus size models	
	Lizzo	
	Joann	
	Spencer Barbosa	
	TikTok trends	
	Depending on platform	
Effectiveness	Positive	Third person effect
		Effect self-image
		Social comparison
		Motivational
		Desire
	Negative	Pushy
		Promoting obesitas
		Too extreme
		Envy
Gendered interpretations	Mostly among females	
	Different content	
	Different needs	
	Taboe among males	

## Data Availability

The data that support the findings of this study are available from the corresponding author, upon reasonable request, as the study involved data derived from focus groups with minors.
